# Impact of vancomycin use trend change due to the availability of alternative antibiotics on the prevalence of *Staphylococcus aureus* with reduced vancomycin susceptibility: a 14-year retrospective study

**DOI:** 10.1186/s13756-022-01140-9

**Published:** 2022-08-05

**Authors:** Yu Ri Kang, Si-Ho Kim, Doo Ryeon Chung, Jae-Hoon Ko, Kyungmin Huh, Sun Young Cho, Cheol-In Kang, Kyong Ran Peck

**Affiliations:** 1 Division of Infectious Diseases, Department of Internal Medicine, Samsung Medical Center, Sungkyunkwan University School of Medicine, 81 Irwon-ro, Gangnam-gu, Seoul, 06351 Republic of Korea; 2 Asia Pacific Foundation for Infectious Diseases, Seoul, Republic of Korea; 3 Division of Infectious Diseases, Samsung Changwon Hospital, Sungkyunkwan University School of Medicine, Changwon, Republic of Korea; 4 Center for Infection Prevention and Control, Samsung Medical Center, Seoul, Republic of Korea

**Keywords:** Mutation, Single-nucleotide polymorphism, Sequence analysis, Data warehouse, Rifampin

## Abstract

**Background:**

We investigated the trend change in vancomycin-intermediate *Staphylococcus aureus* (VISA)/heterogeneous VISA (hVISA) prevalence among methicillin-resistant *S. aureus* (MRSA) bacteremia strains and antistaphylococcal antibiotic use together with mutation studies of vancomycin resistance-related gene loci to evaluate the impact of changes in antibiotic use after new antistaphylococcal antibiotics became available.

**Methods:**

Among 850 healthcare-associated MRSA isolates from 2006 to 2019 at a tertiary hospital in South Korea, hVISA/VISA was determined by modified PAP/AUC analysis, and the identified hVISA/VISA strains were genotyped. Gene mutations at *vraSR, graSR, walKR*, and *rpoB* were studied by full-length sequencing. Antistaphylococcal antibiotic use in 2005–2018 was analyzed.

**Results:**

Two VISA and 23 hVISA strains were identified. The prevalence rate ratio of hVISA/VISA carrying mutations at the two-component regulatory systems among MRSA was 0.668 (95% CI 0.531–0.841; *P* = 0.001), and the prevalence rate ratio of hVISA/VISA carrying *rpoB* gene mutations was 1.293 (95% CI 0.981–1.702; 174 *P* = 0.068). Annual vancomycin use density analyzed by days of therapy (DOT) per 1,000 patient-days did not decrease significantly, however the annual average length of time analyzed by the number of days vancomycin was administered for each case showed a significantly decreasing trend.

**Conclusions:**

During the 14-year period when the average length of vancomycin therapy decreased every year with the availability of alternative antibiotics, the prevalence of hVISA/VISA did not decrease significantly. This seems to be because the resistant strains carrying the *rpoB* mutations increased despite the decrease in the strains carrying the mutations at the two-component regulatory systems.

**Supplementary Information:**

The online version contains supplementary material available at 10.1186/s13756-022-01140-9.

## Introduction

Since *Staphylococcus aureus* with reduced vancomycin susceptibility has emerged, successful treatment with vancomycin for methicillin-resistant *S. aureus* (MRSA) infection has been challenging [1, 2]. Reduced vancomycin susceptibility could present in the whole MRSA population (vancomycin-intermediate *S. aureus*, VISA) or subpopulations (heterogeneous VISA, hVISA), and VISA/hVISA infections frequently have been associated with vancomycin failure or persistent infection [2–5].

The VISA/hVISA phenotypes are associated with mutations in the *vraSR* (vancomycin resistance-associated sensor/regulator), *graSR* (glycopeptide resistance-associated sensor/regulator), and *walKR* (sensor protein kinase/regulator) genes of two-component systems that function during cell-wall synthesis [6, 7]. Mutations in the rifampin resistance-determining region of *rpoB* have also been reported to be associated with emergence of VISA/hVISA [8, 9]. Additionally, prolonged exposure to vancomycin was associated with VISA/hVISA phenotype and cell-wall thickening caused by these mutations [2, 10, 11].

The prevalence of VISA and hVISA over the past decade has increased [5, 12, 13]; however, prevalence varies by region, country, and medical institution [13–15]. Furthermore, antistaphylococcal agents that can replace vancomycin, including linezolid, daptomycin, and tigecycline, have been available since 2002, and studies on how reduction in vancomycin use affected the prevalence of VISA/hVISA are lacking.

In this study, we investigated the trend change in VISA/hVISA prevalence among MRSA bacteremia strains and antistaphylococcal antibiotic use together with mutation studies of vancomycin resistance-related gene loci to evaluate the impact of changes after new antistaphylococcal antibiotics became available.

## Materials and methods

### Bacterial strains and susceptibility testing

We collected all MRSA blood isolates from 2006 to 2019 at Samsung Medical Center (SMC, Seoul, Korea), a large tertiary referral hospital at which more than 70% of patients were referred from other regions across the country. If MRSA were isolated multiple times from one patient, the first MRSA isolate was collected. A total of 984 non-duplicate MRSA isolates were collected. Hospital-onset strain was defined as strain collected from a positive blood culture taken at least 48 h after admission to hospital. Community-onset strain was further classified as SMC-specific healthcare-associated (HCA), HCA (other facilities), or community-associated. SMC-specific HCA infection was defined as an infection that meets any of the following: hospitalization or surgery at SMC within the year preceding the admission; history of hemodialysis at SMC within 30 days before admission; history of catheterization at SMC within 30 days before admission. Finally 850 SMC-associated MRSA strains were included (Additional file 1: Fig. S1).

In vitro antimicrobial susceptibility testing was performed by a broth microdilution method according to Clinical and Laboratory Standards Institute (CLSI) guidelines [16]. *S. aureus* ATCC 29213 and *Enterococcus faecium* ATCC 29212 were used as control strains. This study was approved by the Institutional Review Board (IRB) of SMC.

### VISA/hVISA screening and confirmation

All isolates were screened for VISA or hVISA strains as previously described [17]. Four 10-µl droplets from 0.5 McFarland suspensions were dropped onto brain heart infusion (BHI) agar supplemented with 4 µg/ml (BHI-V4 medium) vancomycin. Plates were incubated for 24–48 h at 37ºC, and individual colonies in each droplet were counted. An isolate was considered VISA/hVISA if at least one droplet contained two or more colonies.

Modified population analysis profile/area under the curve (PAP/AUC) analysis was performed as described previously [17, 18]. Briefly, overnight cultures were grown in trypton soy broth and diluted in saline to 10^–2^ and 10^–5^. Each dilution was aliquoted using a spiral dispenser (Interscience, St. Nom, France) onto BHI agar plates containing 0, 0.5, 1, 2, 3, and 4 mg/L vancomycin. Colonies were counted after a 48-h incubation at 37ºC. Bacterial colony counts were determined using the Scan 500 (Interscience). PAP/AUC was calculated using GraphPad Prism 5.0 (GraphPad Software, La Jolla, CA, USA). The AUC_test_/AUC_Mu3_ ratio was calculated, and strains were determined to be vancomycin-susceptible *S. aureus* (VSSA), hVISA, or VISA according to ratio: VSSA, < 0.9; hVISA, 0.9–1.3; VISA, > 1.3. Mu3 strain (hVISA, ATCC700698), Mu50 (VISA, ATCC700699), and *S. aureus* ATCC29213 (VSSA) were used as controls.

### Genotypic characterization of hVISA/VISA

The SMC-associated hVISA/VISA strains were characterized by multilocus sequence typing (MLST), Staphylococcal Cassette Chromosome *mec* (SCC*mec*) typing, *spa* typing, *agr* typing, and pulsed-field gel electrophoresis (PFGE). MLST was carried out, and sequence types (STs) were assigned by reference to the *S. aureus* MLST website (http://saureus.mlst.net) [19].The SCC*mec* type was profiled by multiplex PCR assay [20]. The *spa* type was determined by PCR sequencing of the repeat region of the *S. aureus* protein, as described previously [21]. The *agr* specificity group was determined by PCR using specific primers as described previously [22]. Clonal relationships were determined by PFGE after DNA digestion by *Sma*I, as previously described [23]. PFGE patterns were analyzed with GelCompar version 6.6 (Applied Maths, Austin, Texas, USA) using the Dice coefficient and were represented by the unweighted pair-grouping method using arithmetic averages (UPGMA) with 1.7 tolerance and 1.2% optimization settings. Results were interpreted using a cut-off point of 80%.

### Mutation detection in two-component regulatory systems and *rpoB* gene

Full-length forward and reverse sequences were obtained for *vraSR*, *graSR, walKR,* and *rpoB* from PCR-amplified fragments using the primers shown in Additional file 2: Table S1 [10, 24, 25]. Sequences were aligned and compared to the reference genome N315 (GenBank accession number *BA000018*). Sequence data were analyzed using EDITSEQ and MEGALIGN software (DNASTAR, Inc., Madison, WI, USA).

### Analysis of antibiotic use

Antibiotic use density rates of vancomycin, teicoplanin, linezolid, and tigecycline for the period from 2005–2018 were calculated annually as days of therapy (DOT)/1,000 patient-days, an index provided by the hospital’s data warehouse for antibiotic use monitoring: SMC Antibiotic Use Guard (SMC ANTIBUG). Additionally, the number of consecutive prescription days or length of therapy (LOT) of vancomycin per prescription event was calculated to estimate the burden of continuous vancomycin exposure. Because patients with decreased creatinine clearance were not administered vancomycin daily, an interval of ≤ 4 days between doses of vancomycin was considered as the same prescription event.

### Statistical analyses

Annual prevalence rates of hVISA/VISA among MRSA were analyzed using Poisson regression. Prevalence rates of hVISA/VISA strains carrying mutations at the two-component systems among MRSA and the strains carrying *rpoB* gene mutation were also determined, respectively. Linear regression analysis was performed to evaluate trend change in antimicrobial use over time, with year as the independent variable. All *P*-values were two-tailed, and *P* < 0.05 was considered statistically significant. All statistical analyses were performed using R software version 3.4.4 (Vienna, Austria; http://www.R-project.org).

## Results

### Changes in prevalence of hVISA/VISA over time

**Fig. 1:**
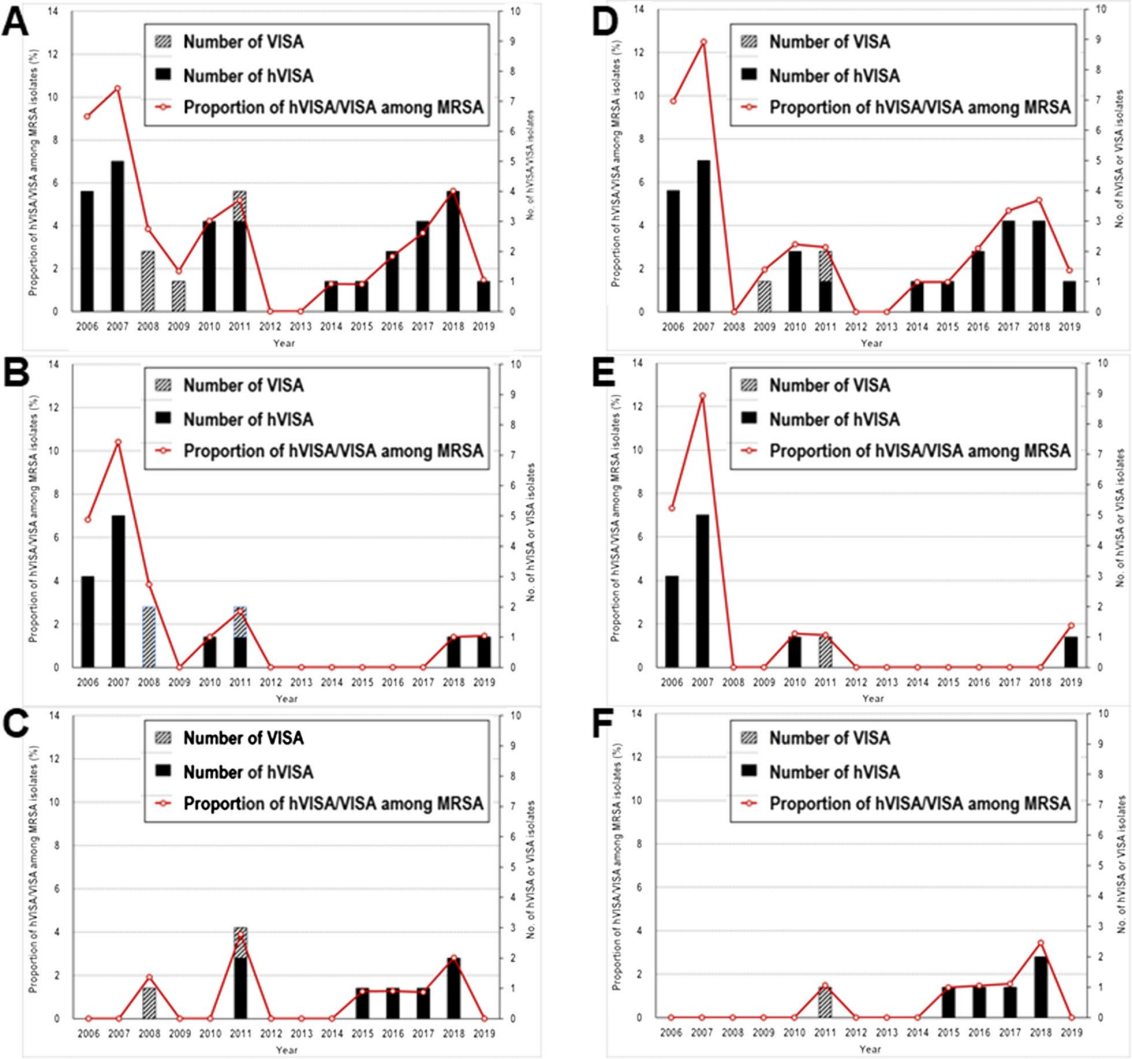
Annual trend in number of hVISA/VISA and hVISA/VISA proportions among MRSA blood isolates. **a** Analysis of all 984 MRSA strains of bacterial collection. **b** hVISA/VISA carrying mutations at the two-component regulatory systems among 984 MRSA strains. **c** hVISA/VISA carrying mutations at the *rpoB* gene loci among 984 MRSA strains. **d** analysis of 850 strains of MRSA related to the Samsung Medical Center. **e** hVISA/VISA carrying mutations at the two-component systems among 850 MRSA strains. **f** hVISA/VISA carrying mutations at the *rpoB* gene loci among 850 MRSA strains. VISA, vancomycin-intermediate *Staphylococcus aureus*; hVISA, heterogeneous VISA; MRSA, methicillin-resistant *S. aureus*

When all 984 MRSA strains were included for analysis, 4 (0.4%) were VISA and 27 (2.7%) were hVISA. The annual prevalence rate of hVISA/VISA decreased by 10% {prevalence rate ratio 0.907 [95% confidence interval (CI) 0.823–0.997; *P* = 0.042]} (Fig. 1a). The prevalence rate ratio of hVISA/VISA carrying mutations at the two-component regulatory systems among MRSA was 0.732 (95% CI 0.619–0.868; *P* < 0.001), and the prevalence rate ratio of hVISA/VISA carrying *rpoB* gene mutations among MRSA was 1.066 (95% CI 0.890–1.276; *P* = 0.488) (Fig. 1b, c). When only 850 SMC-associated MRSA strains were analyzed, the annual prevalence rate ratio of hVISA (23 strains)/VISA (two strains) among MRSA was 0.923 (95% CI 0.830–1.027; *P* = 0.142) (Fig. 1d). The prevalence rate ratio of hVISA/VISA carrying mutations at the two-component systems among MRSA was 0.668 (95% CI 0.531–0.841; *P* = 0.001), and the prevalence rate ratio of hVISA/VISA carrying *rpoB* gene mutations among MRSA was 1.293 (95% CI 0.981–1.702; *P* = 0.068) (Fig. 1e, f).

None of the 25 hVISA/VISA strains were resistant to linezolid and tigecycline, and the rates of resistance to ciprofloxacin, clindamycin, erythromycin, tetracycline, and gentamicin were 92.0%, 80.0%, 92.0%, 84.0%, and 76.0%, respectively. Resistance rates to rifampin and trimethoprim-sulfamethoxazole were 24.0% and 24.0%, respectively (Additional file 2: Table S2).

### Genotypic characteristics of hVISA/VISA strains

Among 25 SMC-associated hVISA/VISA strains from 2006–2019, the most frequent genotype was ST5-SCC*mec* II (n = 20, 80.0%), followed by ST72-SCC*mec* IVA (n = 4, 16.0%) and ST239-SCC*mec* III (n = 1, 4.0%; Table 1, Additional file 3: Fig. S2). Among ST5 hVISA/VISA strains, the most frequent *spa* type was t2460 (n = 15, 75.0%), followed by t002 (n = 3, 15.0%) and t9353 (n = 2, 10.0%). There were three *spa* types in ST72-SCC*mec* IVA: t324 (n = 2, 50.0%), t148 (n = 1, 25.0%), and t664 (n = 1, 25.0%). Analysis of the PFGE band patterns for the 25 strains revealed 11 pulsotypes, with a Dice coefficient cut off of 80% (Additional file 3: Fig. S2). Even strains of the same pulsotype did not belong to cases that occurred at the same time in the same ward.

**Table 1:** Molecular characterization of *S. aureus* strains with reduced vancomycin susceptibility from 2006–2019

Strain ID	Year	VISA/hVISA	MLST	SCC*mec* type	*spa* type	agr	PFGE pulsotype	Mutation sites found						
								*vraR*	*vraS*	*graR*	*graS*	*walR*	*walK*	*rpoB*
K01-SAU-06-1583	2006	hVISA	ST72	IVA	t324	I	P10	E59D	–	–	T224I	–	–	–
K01-SAU-06-1590	2006	hVISA	ST5	II	t002	II	P1	A113V	–	–	–	–	–	–
K01-SAU-06-1591	2006	hVISA	ST5	II	t002	II	P4	–	–	–	–	–	–	–
K01-SAU-06-1613	2006	hVISA	ST5	II	t002	II	P1	A113V	–	–	–	–	–	–
K01-SAU-07-1192	2007	hVISA	ST5	II	t2460	II	P1	A113V	–	–	–	–	–	–
K01-SAU-07-1193	2007	hVISA	ST239	III	t037	I	P7	E59D	–	D148Q	L26F, I59L, T224I	–	A468T	–
K01-SAU-07-1208	2007	hVISA	ST5	II	t2460	II	P1	A113V	–	–	–	–	–	–
K01-SAU-07-1209	2007	hVISA	ST5	II	t2460	II	P3	–	E99K, E117G	–	–	–	–	–
K01-SAU-07-1211	2007	hVISA	ST5	II	t2460	II	P2	E87A, A113V	–	–	–	–	–	–
K01-SAU-09-9664	2009	VISA	ST5	II	t2460	II	P11	–	–	–	–	–	–	–
K01-SAU-10-447	2010	hVISA	ST72	IVA	t148	NT	P8	E59D	–			–	–	–
K01-SAU-10-476	2010	hVISA	ST5	II	t2460	II	P11	–	–	–	–	–	–	–
K01-SAU-11-095	2011	hVISA	ST5	II	t2460	II	P11	–	–	–	–	–	–	–
K01-SAU-11-298	2011	VISA	ST72	IVA	t324	I	P8	E59D, E127K	–	–	T224I	–	–	H481Y
K01-SAU-14-050	2014	hVISA	ST5	II	t2460	II	P11	–	–	–	–	–	–	–
K01-SAU-15-061	2015	hVISA	ST5	II	t2460	II	P11	–	–	–	–	–	–	T518S*
K01-SAU-16-107	2016	hVISA	ST72	IVA	t664	I	P9	–	–	–	–	–	–	–
K01-SAU-16-135	2016	hVISA	ST5	II	t2460	II	P11	–	–	–	–	–	–	A477D
K01-SAU-17-044	2017	hVISA	ST5	II	t2460	II	P6							A477D
K01-SAU-17-072	2017	hVISA	ST5	II	t9353	II	P6							
K01-SAU-17-144	2017	hVISA	ST5	II	t9353	II	P6							
K01-SAU-18-012	2018	hVISA	ST5	II	t2460	II	P7							P475S
K01-SAU-18-025	2018	hVISA	ST5	II	t2460	II	P7							A477D
K01-SAU-18-066	2018	hVISA	ST5	II	t2460	II	P3							
K01-SAU-19-184	2019	hVISA	ST5	II	t2460	II	P5						A400V	

### Frequency of single-nucleotide polymorphism (SNP) in *vraSR*, *graSR*, *walKR*, and *rpoB*

**Fig. 2:**
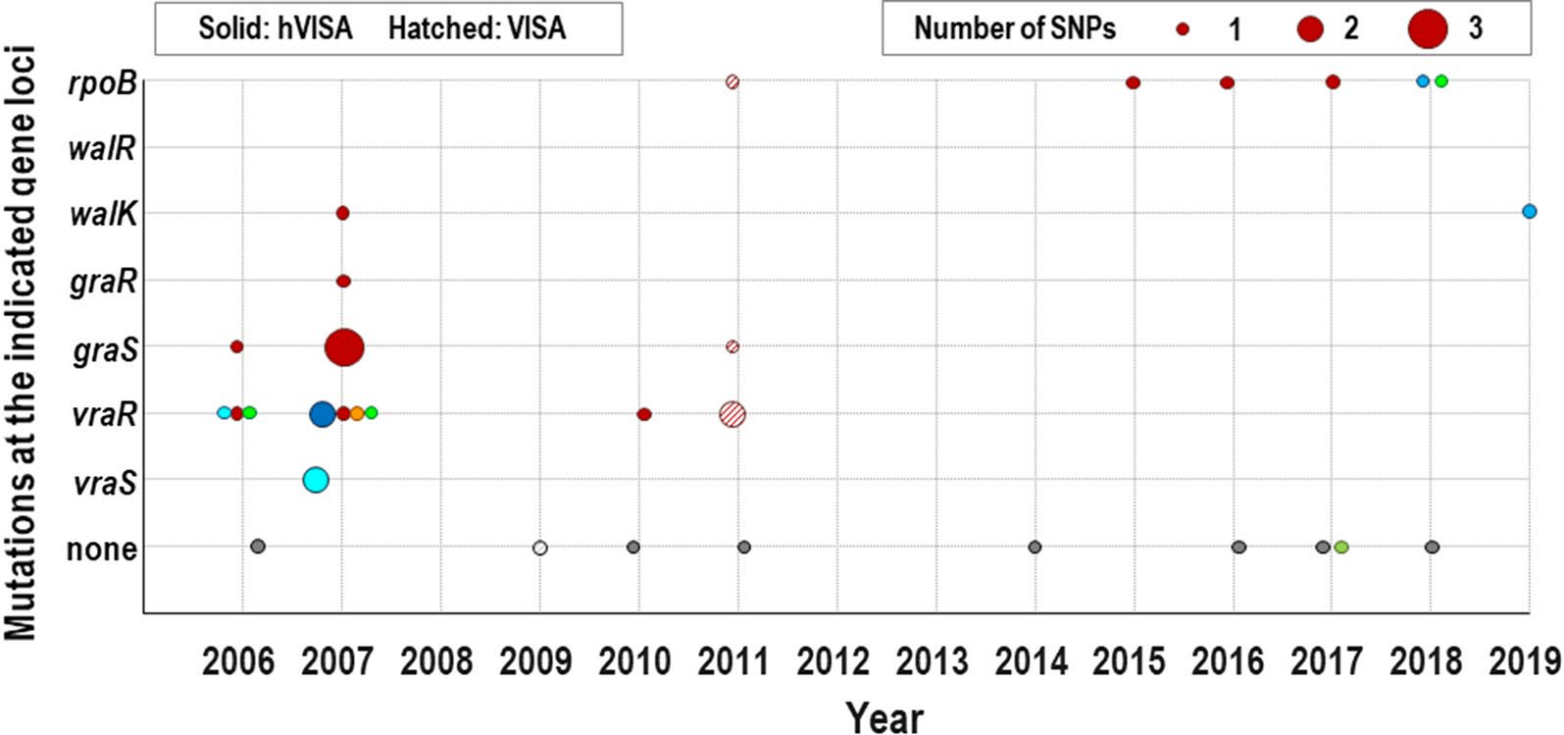
Mutations at the two-component systems and *rpoB* gene loci of 25 hVISA/VISA strains related to the Samsung Medical Center. Circles of the same color on the same vertical line indicate data of the same strain

Multiple SNPs were identified in *vraSR*,* graSR*,* walKR*, and *rpoB* (Table 1). Mutations of the two-component regulatory systems or *rpoB* loci were confirmed in 16 (64.0%) of 25 hVISA/VISA strains. One of two VISA strains showed distinct mutations in *vraR* (E59D, E127K), *graS* (T224I)*,* and *rpoB* (H481Y)*.* Among hVISA, 10 of 23 strains (43.5%) showed distinct mutations in *vraR* (A113V, E59D, E87A), *vraS* (E99K, E117G), *graR* (D148Q)*, graS* (I59L, L26F, T224I)*,* and *walK* (A468T, A400V). Mutations in *rpoB* (A477D, H481Y, P475S, T518S) were found in 6 of 23 hVISA strains (26.1%)*.* Mutations were observed most frequently in *vraSR* loci, followed by *rpoB* and *graSR* loci. Multiple mutations of the *vraSR*, *graSR*, and *walKR* loci were observed only in hVISA/VISA strains until 2011, whereas mutations in *rpoB* were observed only in hVISA/VISA strains collected in 2011 and later (Fig. 2).

### Time trends of antimicrobial use

**Fig. 3:**
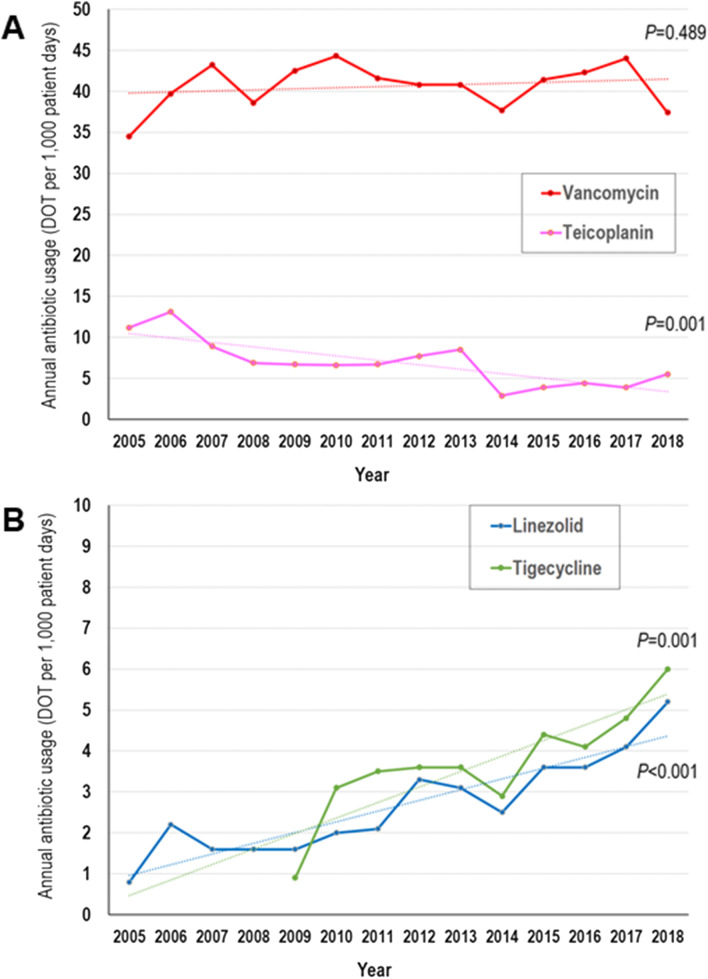
Annual trend of antistaphylococcal antibiotic use density. DOT, days of therapy. **a** vancomycin and teicoplanin. **b** linezolid and tigecycline

**Fig. 4:**
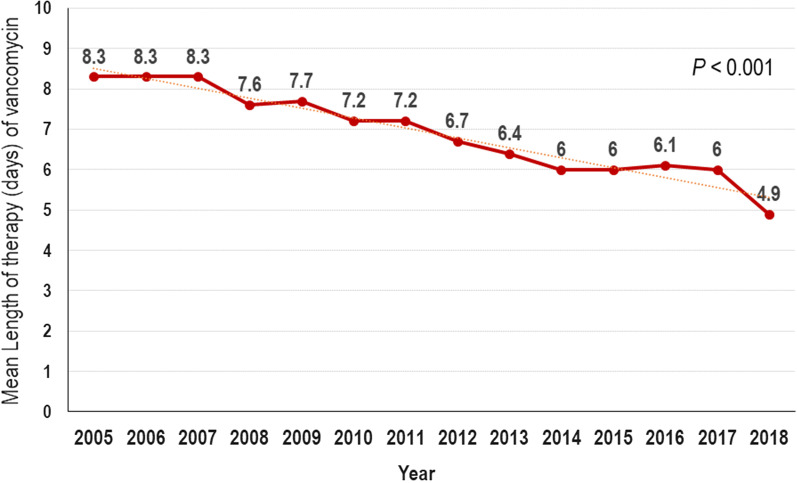
Annual trend in average length of vancomycin therapy

During the study period (2005–2018), annual vancomycin use density in DOT/1,000 patient-days did not show any trend (Fig. 3a). Although annual teicoplanin use showed a significantly decreasing trend (*P* = 0.001), annual glycopeptide use did not. In contrast, annual linezolid and tigecycline use, which could be prescribed alternatively for infections by Gram-positive bacteria including MRSA, showed a significantly increasing trend during the study period (Fig. 3b). In contrast to the vancomycin use density calculated by DOT/1,000 patient-days, annual average length of vancomycin therapy in individual cases showed a significantly decreasing trend over time (Fig. 4).

## Discussion

Our study revealed that annual hVISA/VISA prevalence rates among MRSA bacteremia strains significantly decreased during the 14-year period, however the prevalence rates did not show a significant decrease when limited to the strains only from the SMC-specific HCA infections, except for cases transferred from the other hospitals/chronic care facilities and community-associated cases. Instead, subgroup analysis showed that only the hVISA/VISA strains showing the mutations in the two-component regulatory systems showed a significant decrease, whereas the *rpo-B* mutant strain tended to increase. In particular, there was no VISA strain after 2011 and *rpo-B* mutant strain was only found since 2011. Analysis of antibiotic use data showed that the average length of vancomycin therapy during the study period significantly decreased every year, suggesting that this decrease might have affected the decrease of hVISA/VISA, especially strains with mutations in the two-component systems.

The previous multicenter study in South Korea reported that hVISA prevalence among MRSA from six medical centers in South Korea decreased from 25.0% during 2006–2007 to 2.2% during 2011–2013 [15]. One tertiary hospital in South Korea reported 37.7% hVISA prevalence during 2008–2010 [14]; however, the high prevalence here might be associated with clonal dissemination. In contrast, we posit that our findings were not affected by clonal dissemination because the PFGE analysis of hVISA/VISA strains showed various pulsotypes. Although some strains belonged to the same pulsotype, the time of bacteremia was different or infection occurred in a different ward, suggesting that it was not a case due to direct transmission or outbreak.

As vancomycin substitutes became available, it was predicted that vancomycin use would decrease; however, the annual vancomycin use density analyzed by DOT/1,000 patient-days in our study did not decrease significantly. Although teicoplanin use density showed a significant decrease, the amount of teicoplanin used was relatively lower than that of vancomycin, so the trend did not show a significant change when analyzed by overall use density of glycopeptide. On the other hand, linezolid and tigecycline use showed a significantly increasing trend. Daptomycin was not available in South Korea until 2020, and was not included in this study. The results of our study in which vancomycin use density did not decrease, but the annual average LOT analyzed by the number of days vancomycin was administered for each case showed a significantly decreasing trend are very interesting. This was a convincing result considering that prolonged exposure to vancomycin is the most important risk factor for emergence of VISA or hVISA [2, 3].

The possible reasons for the results that the annual vancomycin use density (DOT/1,000 patient-days) did not decrease despite the decrease in annual average LOT of vancomycin are as follows. First, improved hand hygiene compliance and enhanced infection control have contributed to reducing the incidence of MRSA infection in hospitals, but MRSA remains the major causative organism of healthcare-associated infections in Korean hospitals [26, 27]. Second, despite the introduction of an alternative antibiotics to vancomycin, the standard treatment recommended for MRSA infection is still vancomycin or teicoplanin in South Korea, which has a universal health insurance system. For these reasons, annual vancomycin use density does not appear to have decreased significantly. On the other hand, the decrease in the average LOT in individual cases is thought to be due to antimicrobial stewardship activities by infection specialists who recommends changing to an alternative antibiotic early in case that shows poor clinical response to vancomycin or adverse drug reactions. In addition, SMC Computerized Antimicrobial Stewardship System (SMC COMPASS) which was designed to automatically stop prescription if it does not receive approval by an infection specialist within 2 days after prescribing vancomycin, may also have contributed to the decrease in LOT of vancomycin. Teicoplanin was more likely to be used as an alternative to vancomycin in Korea [28], but as linezolid became available, teicoplanin use decreased.

Considering that the hVISA/VISA phenotype is associated with mutations at the *vraSR*, *graSR*, and *walKR* loci due to prolonged exposure to vancomycin [2, 10, 11], our findings that the annual prevalence rate of hVISA/VISA carrying mutations at these two-component systems among MRSA showed a significantly decreasing trend, and multiple mutations of these loci were observed only in hVISA/VISA strains until 2011 could be explained by the finding that the length of vancomycin therapy decreased following the availability of vancomycin-replacing antibiotics in the hospital.

Mutations at the *rpoB* locus were found in six hVISA/VISA strains, and mutations (A477D, H481Y) were confirmed in four of six rifampin-resistant hVISA/VISA strains. Interestingly, the *rpoB* gene mutation continued to be frequently observed in contrast to the mutations at the two-component systems were rarely observed in the later study period. Analysis of the annual prevalence rate ratio of hVISA/VISA carrying *rpoB* gene mutations among SMC-associated MRSA also tended to increase. Although this study did not include an analysis of rifampin use, these findings are corroborated by the increasing need to administer rifampin for MRSA infections. Because the occurrence of device-related infections is increasing, and rifampin, which has excellent biofilm penetration, has been recommended as an adjunctive therapy for *S. aureus* infection [29], it is possible that mutation of the *rpoB* locus and induction of rifampin resistance can lead to hVISA/VISA development [30]. Despite the decline in hVISA/VISA prevalence due to reduced exposure to long-term use of vancomycin, this appears to have led to low but continued hVISA prevalence in our study.

The main strength of this study is that it is, to our knowledge, the first to analyze the trend change in hVISA/VISA prevalence among MRSA bacteremia strains and antistaphylococcal antibiotic use together with mutation studies of vancomycin resistance-related gene loci. In order to properly determine the impact of changes in the use of antibiotics in our hospital on the trend in hVISA/VISA prevalence rates, only cases associated with our hospital were included, and all cases that were transferred from other hospitals or chronic care facilities and that were community-associated were excluded. Additionally, high-quality antibiotic use data from the hospital data warehouse were analyzed to identify trends in hVISA/VISA occurrence in connection with antibiotic use over a long study period to evaluate the impact of changes in antibiotic use after new antistaphylococcal antibiotics became available.

Our study has some limitations. First, this was a retrospective study from a single medical center, which limits the generalizability of our findings. Second, because we only investigated hVISA/VISA phenotypes among MRSA bacteremia cases, hVISA/VISA strains that could emerge from non-bacteremic cases were not included. Third, since no mutation in the *vraSR*, *graSR*, *walKR*, and *rpoB* loci was found in the 9 of 25 hVISA/VISA strains, there may be other vancomycin resistance mechanisms, which may also limit the interpretation of this study. Fourth, because the analysis of antistaphylococcal antibiotic use was limited to a few specific antibiotics, the effect of changes in other antibiotics was not considered. In particular, analysis of trend change in rifampin use could have provided valuable information, but these data were excluded from the analysis because it represented an overall amount used to treat tuberculosis as well as the amount used to treat *S. aureus* infection. Lastly, in this study, improvement of hand hygiene of the healthcare workers and reinforcement of infection control strategies in hospital which may also have influenced the change in the prevalence of hVISA/VISA were not considered. However, the investigation of 25 hVISA/VISA strains did not show cases suspected of a small outbreak or direct transmission.

## Conclusions

Annual prevalence rates of hVISA/VISA among healthcare-associated MRSA bacteremia strains did not decrease during the 14-year period in a tertiary care hospital in South Korea. However, the subgroup analysis revealed that annual prevalence rates of hVISA/VISA carrying mutations at the two-component systems among healthcare-associated MRSA bacteremia strains significantly decreased during the study period. The average length of vancomycin therapy decreased every year as alternative antibiotics became available, which may have had an effect on the decrease in the hVISA/VISA prevalence. The increase in hVISA carrying *rpoB* mutation in the later study period offset the decrease in hVISA/VISA prevalence as the vancomycin use decreased. The increasing need to treat MRSA infection with rifampin and the fact that hVISA/VISA can emerge from *rpoB* mutation require continued surveillance for hVISA/VISA phenotypes and antibiotic use monitoring.

## Supplementary Information

**Additional file 1: Fig. S1**. Flow diagram showing strategies for selection of bacterial strains.

**Additional file 2. Table S1**. Primer sequences for amplification of two-component systems and *rpoB* gene loci. **Table S2**. Phenotypic characterization of 25 *S. aureus* strains with reduced vancomycin susceptibility.

**Additional file 3: Fig. S2**. Genotypic characteristics of 25 hVISA/VISA strains associated with the Samsung Medical Center. VISA, vancomycin-intermediate *Staphylococcus aureus*; hVISA, heterogeneous VISA; CO-HCA, community-onset healthcare-associated; HO, hospital-onset; F, floor; H, hospital; ICU, intensive care unit; MICU, medical ICU; SICU, surgical ICU.

## Data Availability

The datasets generated and/or analysed during the current study are not publicly available, but are available from the corresponding author on reasonable request.
