# Supplementary figures and images for: Impact of vancomycin use trend change due to the availability of alternative antibiotics on the prevalence of Staphylococcus aureus with reduced vancomycin susceptibility: a 14-year retrospective study
Source: Antimicrob Resist Infect Control. 2022 Aug 5;11:101. doi: 10.1186/s13756-022-01140-9 (PMC9354315; doi:10.1186/s13756-022-01140-9)

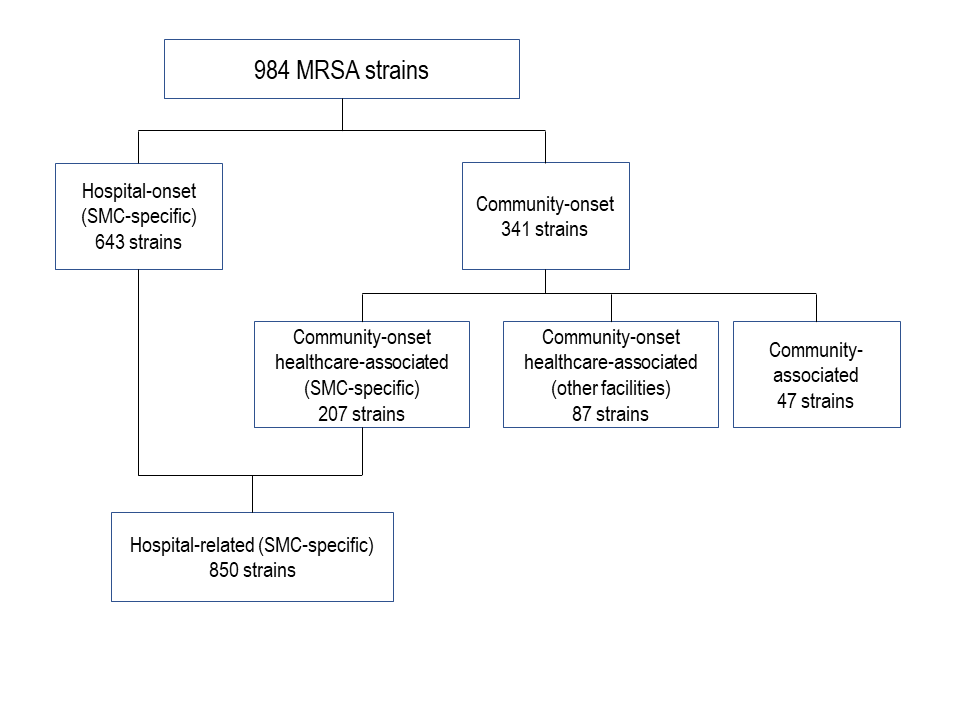

Supplement: Supplementary file 1 — Additional file 1: Fig. S1. Flow diagram showing strategies for selection of bacterial strains. [file 13756_2022_1140_MOESM1_ESM.tif]

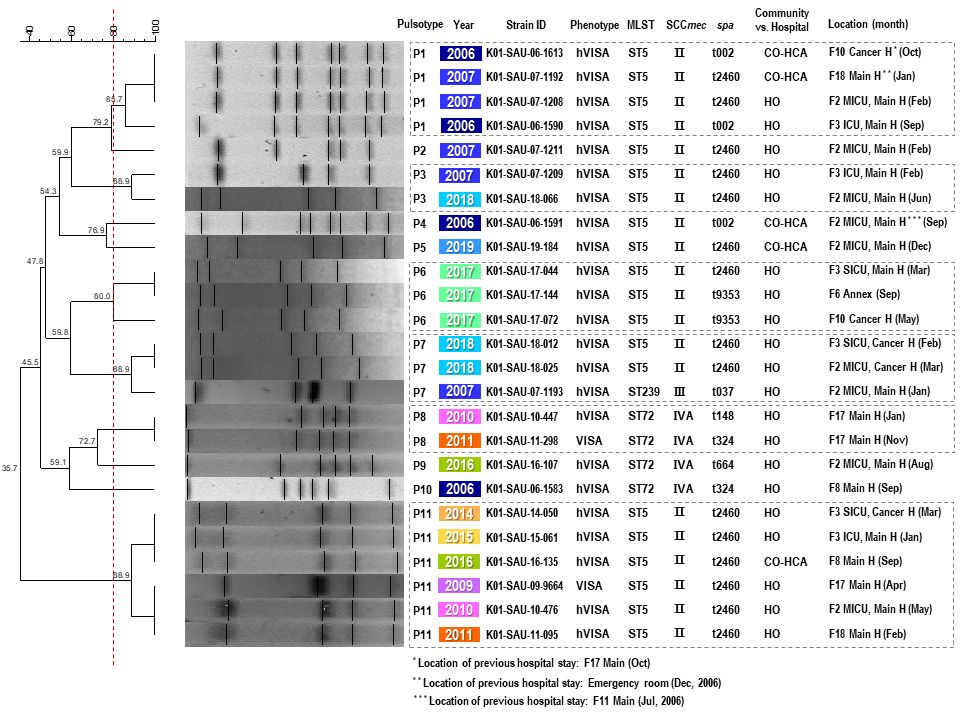

Supplement: Supplementary file 3 — Additional file 3: Fig. S2. Genotypic characteristics of 25 hVISA/VISA strains associated with the Samsung Medical Center. VISA, vancomycin-intermediate Staphylococcus aureus; hVISA, heterogeneous VISA; CO-HCA, community-onset healthcare-associated; HO, hospital-onset; F, floor; H, hospital; ICU, intensive care unit; MICU, medical ICU; SICU, surgical ICU. [file 13756_2022_1140_MOESM3_ESM.tif]
