# Supplementary material for: Impact of vancomycin use trend change due to the availability of alternative antibiotics on the prevalence of Staphylococcus aureus with reduced vancomycin susceptibility: a 14-year retrospective study
Source: Antimicrob Resist Infect Control. 2022 Aug 5;11:101. doi: 10.1186/s13756-022-01140-9 (PMC9354315; doi:10.1186/s13756-022-01140-9)
Supplement: Supplementary file 2 — Additional file 2. Table S1. Primer sequences for amplification of two-component systems and rpoB gene loci. Table S2. Phenotypic characterization of 25 S. aureus strains with reduced vancomycin susceptibility. [file 13756_2022_1140_MOESM2_ESM.docx]

**Supplementary table 1. Primer sequences for amplification of two-component systems and *rpo*B gene loci**

| Gene | Primer | Sequence | Reference |
| --- | --- | --- | --- |
| *vraR*, *vraS* | vra1F | 5' AGT GAT ACC TAT CCA GAA GAT 3' | Hafer *et al.* (2012) |
|  | vra1656R | 5' TGG TTC GGT ACT CGC ATA CA 3' | Hafer *et al.* (2012) |
|  | vra1571F | 5' CAA CTG TTT CGC CTT CCA TT 3' | Hafer *et al.* (2012) |
|  | vra3199R | 5' GGA TGG TCC GAT TTT AAC GA 3' | Hafer *et al.* (2012) |
| *graR*, *graS* | SA0613F | 5' GCT TTG AAG TTG ACT GCC GGA T 3' | Hafer *et al.* (2012) |
|  | SA0615R | 5' CTA AAA TTG CCA CTT TAA CAC TC 3' | Hafer *et al.* (2012) |
|  | SA0614F2 | 5' GAT GAA GCA TTT GTT AGT GAT AAT 3' | Hafer *et al.* (2012) |
| *walR*, *walk* | walRK_F1 | 5' GCG CAC AAT AAC AAA AAT AG 3' | Hafer *et al.* (2012) |
|  | walRK_R1 | 5' TTA TTA TTC ATC CCA ATC ACC 3' | Hafer *et al.* (2012) |
|  | walRK_F2 | 5' ATT TCC TCC AAC AAC ATG AG 3' | Hafer *et al.* (2012) |
|  | walRK_R2 | 5' GTG ATA CAT TGG CAA CAA ATT 3' | Hafer *et al.* (2012) |
| *rpoB* | rpoB_F1 | 5' GCA AGG TAT GCC ATC TGC AAA G 3' | Wang *et al*. (2017) |
|  | rpoB_R1 | 5' TTG CTT CGG CGA TAC ATC CA 3' | Wang *et al*. (2017) |
|  | rpoB_F2 | 5' ACG TGA ACG TGC TCA AAT GG 3' | Wang *et al*. (2017) |
|  | rpoB_R2 | 5' ATG CCT TTG TAG CGA ACA CG 3' | Wang *et al*. (2017) |

**Supplementary table 2. Phenotypic characterization of 25 *S. aureus* strains with reduced vancomycin susceptibility**

| Strain  ID | Year | VISA/  hVISA | MIC (mg/L), susceptibility | | | | | | | | | |
| --- | --- | --- | --- | --- | --- | --- | --- | --- | --- | --- | --- | --- |
|  |  |  | Penicillin | Oxacillin | Ciprofloxacin | Clinamycin | Erythromycin | Tetracycline | TMP/SMX | Gentamicin | Rifampin | Linezolid |
| K01-SAU-06-1583 | 2006 | hVISA | 32, R | 64, R | 0.25, S | 0.12, S | >64, R | 0.12, S | 0.06/1.18, S | 4, I | 0.015, S | 2, S |
| K01-SAU-06-1590 | 2006 | hVISA | 32, R | >64, R | 16, R | >64, R | >64, R | 32, R | 0.06/1.18, S | >64, R | 0.015, S | 2, S |
| K01-SAU-06-1591 | 2006 | hVISA | 32, R | >64, R | 64, R | >64, R | >64, R | 64, R | 0.06/1.18, S | >64, R | 0.015, S | 2, S |
| K01-SAU-06-1613 | 2006 | hVISA | 32, R | >64, R | 16, R | >64, R | >64, R | 32, R | 0.06/1.18, S | >64, R | 0.015, S | 2, S |
| K01-SAU-07-1192 | 2007 | hVISA | 64, R | >64, R | 32, R | >64, R | >64, R | 64, R | 0.06/1.18, S | 0.5, S | 16, R | 2, S |
| K01-SAU-07-1193 | 2007 | hVISA | 64, R | >64, R | 32, R | >64, R | >64, R | 32, R | 16, 304, R | >64, R | 0.015, S | 2, S |
| K01-SAU-07-1208 | 2007 | hVISA | 64, R | >64, R | 32, R | >64, R | >64, R | 64, R | ≤0.03/0.59, S | >64, R | 0.015, S | 2, S |
| K01-SAU-07-1209 | 2007 | hVISA | 32, R | >64, R | >64, R | >64, R | >64, R | 64, R | ≤0.03/0.59, S | >64, R | 0.015, S | 2, S |
| K01-SAU-07-1211 | 2007 | hVISA | 64, R | >64, R | 64, R | >64, R | >64, R | 64, R | ≤0.03/0.59, S | >64, R | 0.015, S | 2, S |
| K01-SAU-09-9664 | 2009 | VISA | 16, R | >64, R | 64, R | >64, R | >64, R | 32, R | 4/76, R | 0.5, S | 16, R | 2, S |
| K01-SAU-10-447 | 2010 | hVISA | 32, R | 64, R | 16, R | 0.25, S | 1, I | 0.25, S | 32/608, R | 2, S | 0.015, S | 2, S |
| K01-SAU-10-476 | 2010 | hVISA | 32, R | >64, R | >64, R | >64, R | >64, R | 64, R | 16/304, R | >64, R | 0.015, S | 2, S |
| K01-SAU-11-095 | 2011 | hVISA | 32, R | >64, R | >64, R | >64, R | >64, R | 64, R | 0.25/4.75, S | >64, R | 0.015, S | 2, S |
| K01-SAU-11-298 | 2011 | VISA | 32, R | >64, R | 0.5, S | 0.25, S | >64, R | 0.5, S | 0.25/4.75, S | 0.5, S | 16, R | 2, S |
| K01-SAU-14-050 | 2014 | hVISA | 64, R | >64, R | >64, R | >64, R | >64, R | 16, R | 0.25/4.75, S | >64, R | 0.015, S | 2, S |
| K01-SAU-15-061 | 2015 | hVISA | 32, R | >64, R | >64, R | >64, R | >64, R | 64, R | 0.25/4.75, S | >64, R | 0.015, S | 2, S |
| K01-SAU-16-107 | 2016 | hVISA | >64, R | >64, R | 32, R | >64, R | >64, R | 32, R | 8/152, R | 64, R | 0.015, S | 2, S |
| K01-SAU-16-135 | 2016 | hVISA | 64, R | >64, R | 64, R | 0.25, S | 0.5, S | 2, S | 16/304, R | >64, R | 16, R | 2, S |
| K01-SAU-17-044 | 2017 | hVISA | 32, R | >64, R | >64, R | >64, R | >64, R | 64, R | 0.25/4.75, S | >64, R | 16, R | 2, S |
| K01-SAU-17-072 | 2017 | hVISA | 64, R | >64, R | >64, R | >64, R | >64, R | 64, R | 0.25/4.75, S | >64, R | 0.015, S | 2, S |
| K01-SAU-17-144 | 2017 | hVISA | 64, R | >64, R | >64, R | >64, R | >64, R | 64, R | 0.25/4.75, S | >64, R | 0.015, S | 2, S |
| K01-SAU-18-012 | 2018 | hVISA | 32, R | >64, R | >64, R | >64, R | >64, R | 64, R | 0.25/4.75, S | >64, R | 0.015, S | 1, S |
| K01-SAU-18-025 | 2018 | hVISA | 64, R | >64, R | >64, R | >64, R | >64, R | 32, R | 0.25/4.75, S | >64, R | 16, R | 1, S |
| K01-SAU-18-066 | 2018 | hVISA | 32, R | >64, R | >64, R | >64, R | >64, R | 32, R | 0.12/2.37, S | >64, R | 0.015, S | 2, S |
| K01-SAU-19-184 | 2019 | hVISA | 64, R | >64, R | >64, R | 0.25, S | >64, R | 64, R | 0.12/2.37, S | 1, S | 0.015, S | 2, S |
